# Microstructures and Properties of Porous Liquid-Phase-Sintered SiC Ceramic by Hot Press Sintering

**DOI:** 10.3390/ma12040639

**Published:** 2019-02-20

**Authors:** Yajie Li, Haibo Wu, Xuejian Liu, Zhengren Huang

**Affiliations:** 1 State Key Laboratory of High Performance Ceramics and Superfine Microstructure, Shanghai Institute of Ceramics, Chinese Academy of Sciences, Shanghai 200050, China; liyj@shanghaitech.edu.cn (Y.L.); xjliu@mail.sic.ac.cn (X.L.); 2 School of Physical Science and Technology, ShanghaiTech University, Shanghai 201210, China; 3 Suzhou Research Center, Shanghai Institute of Ceramics, Chinese Academy of Sciences, Suzhou 215411, China

**Keywords:** silicon carbide, liquid phase sintering, porosity, microstructure, strength

## Abstract

Porous liquid-phase-sintered SiC (L-SiC) ceramics were successfully fabricated by hot press sintering (HP) at 1800 °C in argon, using Al_2_O_3_ and Y_2_O_3_ as oxide additions. By varying the starting coarse SiC particle size, the relationships between pore microstructures and flexural strength as well as gas permeability of porous L-SiC were examined. All the as-sintered samples possessed homogeneous interconnected pores. The porosity of porous L-SiC decreased from 34.0% to 25.9%, and the peak pore size increased from 1.1 to 3.8 μm as the coarse SiC particle sizes increased. The flexural strengths of porous L-SiC ceramics at room temperature and 1000 °C were as high as 104.3 ± 7.3 MPa and 78.8 ± 5.1 MPa, respectively, though there was a decrease in accordance with their increasing pore sizes and particle sizes. Moreover, their gas permeability increased from 1.4 × 10^−14^ m^2^ to 4.6 × 10^−14^ m^2^ with the increase of pore size in spite of their decreased porosity.

## 1. Introduction

Porous SiC ceramics have been wildly investigated because of their excellent flexural strength, superior chemical and thermal stability, outstanding thermal conductivity, low thermal expansion coefficient, large specific surface area, excellent corrosion resistance, and so on [1,2,3,4,5]. Therefore, porous SiC ceramics are potential materials to be applied in the energy and environment fields for uses such as catalyst supports, gas burner media, high-temperature filters for flue-gas, and volumetric absorbers of solar radiation [1,6,7,8], etc. In the previous reports, there are various studies on the fabrication methods of porous SiC ceramics, including the recrystallization [9], foaming-gel casting [4,10,11,12], gelation-freezing [13,14,15], in-situ reaction [16,17], sacrificing template [18], and sol-gel methods [19,20]. In these fabrication methods, most of the reported porous SiC ceramics were solid-state sintered because of their better heat stability and corrosion resistance as a result of clear grain boundaries and the strong bonding interface between SiC grains [4,10,11,12,14,15]. However, high sintering temperature (up to 2200 °C) and high purity raw materials are the minimum requirements for the fabrication of solid-state-sintered SiC (S-SiC), which directly increase production costs. Compared with S-SiC ceramics, liquid-phase-sintered SiC (L-SiC) ceramics with oxide additions show lower sintering temperatures and better mechanical strength. The liquid phase formed by the oxide between SiC particles can accelerate the sintering rate and effectively reduce sintering temperature to 1800–2000 °C [21].

In porous L-SiC ceramics, the liquid phase is formed around the low eutectic points of the oxide additives and diffuses into the space between the grains [22]. During this process, small SiC particles dissolving into the liquid phase quickly spread and are deposited on large particles. Subsequently, the surface of SiC grains is covered and connected by the liquid phase [1,22]. Thanks to the partial sintering and a tiny bit of oxide additions, the densification of sintered bodies is inadequate, and then pores are successfully formed [23]. Earlier studies about porous L-SiC ceramics have mostly focused on the types of additions such as silica [24,25], alumina [21], yttria [26], silicon nitride [27], mullite [28,29,30], glass frit, cordierite [31], CeO_2_ [16], V_2_O_5_ [32], and so on. They have summarized the effects of starting powders, additive amount, and sintering temperature on the microstructure of porous L-SiC ceramics, which were all prepared by pressureless sintering. Hot press sintering (HP) of porous L-SiC ceramics has not been studied yet.

In our work, porous L-SiC ceramics were prepared by HP using Al_2_O_3_ and Y_2_O_3_ as sintering aids. Pore microstructures of the sintered samples were tailored through varying the size of introduced coarse SiC powder. The dependence of key properties including flexural strength at room temperature and high temperature as well as gas permeability on the porosity, pore size, and microstructure of porous L-SiC ceramics were systematically investigated.

## 2. Experimental Details

### 2.1. Raw Materials

**Figure 1:**
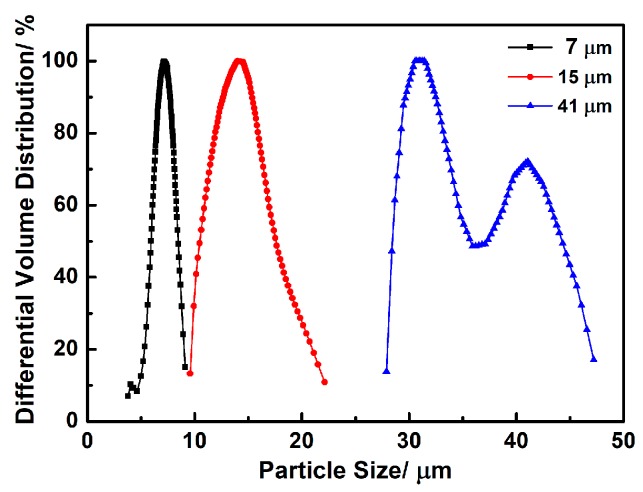
Particle size distribution curves of ~7, ~15, and ~41 μm α-SiC powders.

Commercial fine and coarse α-SiC powders (purity > 99%, Pingdingshan Yicheng New Material Co., Henan, China) were graded and used as the main materials: Fine ones with average particle sizes of ~0.5 μm, and three types of coarse ones with average particle sizes of ~7, ~15 and ~41 μm respectively. The particle size distribution curves of coarse α-SiC powders are shown in Figure 1. The Commercial Al_2_O_3_ powder (~1.5 μm, Fenghe Ceramic Co., Ltd., Shanghai, China) and Y_2_O_3_ powder (~0.8 μm, Dafeng Yuelong Chemical Co., Ltd., Yancheng, China) were added as sintering aids for the porous L-SiC ceramic. Al_2_O_3_/Y_2_O_3_ powders were directly used without any treatment and pre-mixed according to the mass ratio of 78:97 before the preparation of porous L-SiC ceramics. 

### 2.2. Processing

Fine SiC powders, coarse SiC powders, and the mixture of Al_2_O_3_ and Y_2_O_3_ powders were mixed at the mass ratio of 10:90:6 in ethanol and milled with SiC balls for 4 h at 300 r·min^−1^ to prepare the homogeneous suspension. After milling, the suspension was dried using an oven at 60 °C for more than 12 h, crushed, and screened with a 100-mesh sieve. At last, the dried powder was placed in a carbon mold and hot press sintered at 1800 °C for 2 h under a pressure of 30 MPa in argon. In the experiment, the particle size of coarse SiC powder was changed to control the microstructures of prepared porous L-SiC ceramics. The samples containing ~7, ~15, and ~41 μm of coarse SiC powder were marked as L7, L15, and L41, respectively.

### 2.3. Characterization

The immersion densities were measured using the Archimedes method in deionized water, and the relative densities, porosities, and apparent porosities of samples were calculated according to theoretical density. In addition, closed porosity can be gained by subtracting apparent porosity from total porosity. The microstructure and morphology of porous L-SiC ceramics were observed with a scanning electron microscopy (SEM, Phenom ProX, Phenom-World, Eindhoven, The Netherlands), and pore size distribution was measured using mercury intrusion porosimetry (MIP, AutoPoreIV 9510, Micromeritics, Norcross, GA, USA). The Darcy nitrogen permeability was evaluated by a Pore-size Distribution Analyzer (PSDA-20, GaoQ Functional Materials Co., Ltd., Nanjing, China), and the specimens were processed into sheets with the thickness of 3 mm. The flexural strengths of samples at normal temperature and 1000 °C were evaluated by a three-point bending test using a computer-controlled electric universal testing machine (AGS-X, Shimadzu Corp., Kyoto, Japan) with a cross-head speed of 0.5 mm·min^−1^ and a rising temperature rate of 200 °C·min^−1^. The geometrical sizes of samples were 3 mm × 4 mm × 36 mm, and five samples of each composition were tested to determine strength.

## 3. Results and Discussion

### 3.1. Pore Microstructures of Porous L-SiC Ceramics

Pore microstructure is a key influence factor of the properties of porous material. To study the properties of porous L-SiC ceramics, it is necessary to consider the porosity, pore size, and microstructure in advance.

**Figure 2:**
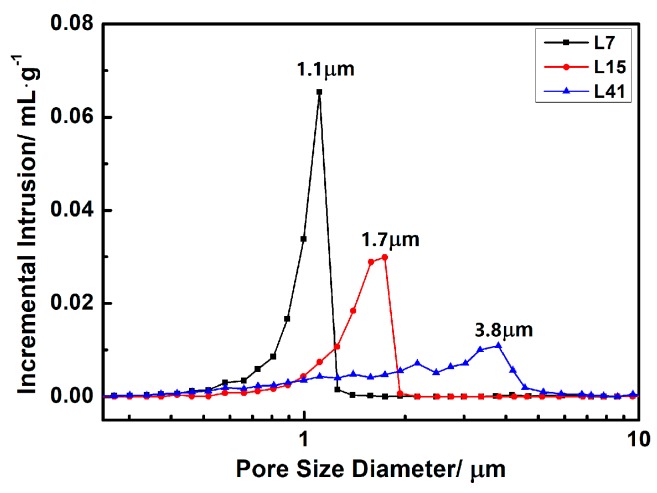
Pore size distribution of porous L-SiC ceramics with different particle sizes of the initial coarse SiC powder.

The relative densities and porosities of porous L-SiC ceramics are listed in Table 1, and the pore size distribution curves of porous L-SiC ceramics are shown in Figure 2. As the coarse particle size increased from ~7 to ~41 μm, the total porosity decreased from 34.0% to 25.9%, and the apparent porosity decreased from 33.5% to 25.8%. This could be ascribed to the filling of space between SiC particles by fine particles owing to the wider size distribution of starting coarse SiC powder. As shown in Figure 2, the peak pore size of the porous L-SiC ceramic increased from 1.1 to 3.8 μm with the increasing size of coarse SiC particles from ~7 to ~41 μm. It indicated that pores with bigger sizes were more likely to be filled or narrowed by the liquid phase during sintering. Moreover, the reduction in the number of pores led to a decrease of total porosity. With the same weight ratio of coarse and fine SiC particles, the samples with smaller sizes of coarse SiC particles had more pores because of the greater number of formed interspaces between particles. Besides, all as-sintered samples exhibited low closed porosities of ≤ 0.5%, illustrating excellent connectivity between the formed pores. In Figure 2, the pore size distribution of the samples with bigger pore sizes was wider and presented two peaks, which was probably induced as well by the wider particle size distribution of the raw coarse SiC powder in Figure 1.

**Figure 3:**
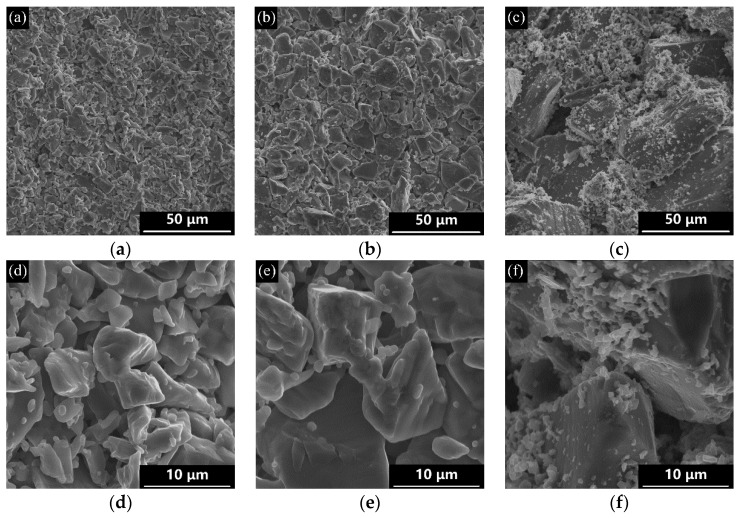
SEM images of porous L-SiC ceramics with different particle sizes of initial coarse SiC powder: (**a**,**d**) 7 μm; (**b**,**e**) 15 μm; (**c**,**f**) 41 μm.

Figure 3 shows the microstructures on the cross section of as-acquired samples. Uniformly distributed pores were successfully formed in the porous L-SiC ceramic. With the increase of the coarse SiC particle size, an increase in pore size could be observed apparently in Figure 3a–c, which coincided well with the result of the pore size distribution in Figure 2. At 1800 °C, Al_2_O_3_ and Y_2_O_3_ were melted into a liquid state and accumulated around the SiC particles, especially near the point of contact between the two particles. In Figure 3d–f, the necks between the SiC grains were formed in all the samples which contributed to the mechanical properties of the porous ceramics. It was commonly observed that the samples with small pores possessed relatively smooth pore walls while the samples with big pores had a large number of irregular pores. Accordingly, it was possible to control the pore microstructure of the porous L-SiC ceramic by changing the particle size of the initial coarse SiC powder.

### 3.2. Flexural Strengths of Porous L-SiC Ceramics

**Figure 4:**
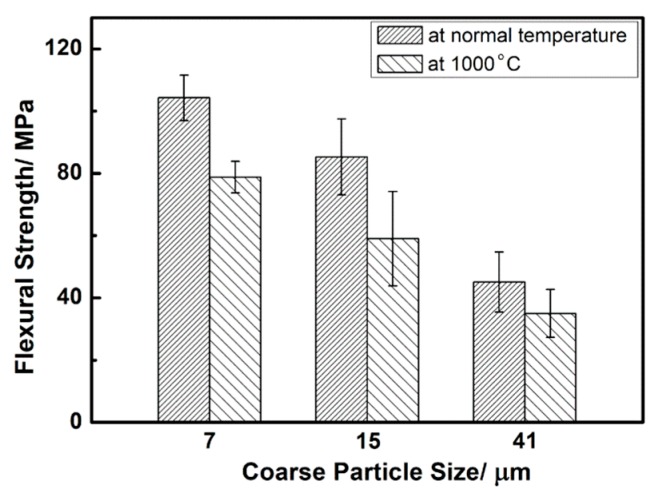
Three-point flexural strengths of porous L-SiC ceramics with different particle sizes of the initial coarse SiC powder at normal temperature and 1000 °C.

**Figure 5:**
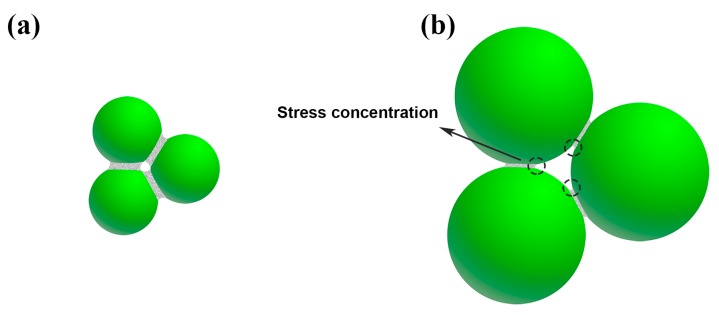
Schematic illustration of the sintering neck in porous L-SiC ceramics from (**a**) small particles and (**b**) large particles.

Figure 4 shows the flexural strength of porous L-SiC ceramics introduced by different particle sizes of coarse SiC powder at normal temperature and at 1000 °C in argon. For sample L7 with a porosity of 34.0%, its flexural strength at normal temperature was as high as 104.3 ± 7.3 MPa and pretty close to Zhao’s result (103 MPa) with the porosity of 35.7% [1], which has the highest flexural strength under the conditions of almost the same porosity compared to the previous studies. Moreover, with the increase of coarse particle sizes, there is a decrease in flexural strength due to the small interparticle-bonding area and irregularity in the pore shape. According to Bukhari’ study [33], small bonding areas between SiC grains formed by the initial powder with the larger particle size reduced the flexural strength of the porous L-SiC ceramic. Figure 5 shows the difference of the neck structures of the porous L-SiC ceramics respectively from small and big SiC particles. Using the same amount of additions, the pores formed between the small particles had smooth continuous pore walls and were shaped like spherical spaces (seen in Figure 3a,c). However, the irregular pores formed between the big particles possessed many points of contact where the concentration of stress appeared during the deformation under the bending test, directly leading to a decrease in flexural strength. Moreover, an increase in pore size would also decrease the strength of the porous L-SiC ceramics [4].

At the temperature of 1000 °C, the flexural strengths of porous L-SiC ceramics also decreased with the increase of coarse particle size. The flexural strengths of L7 (78.8 ± 5.1 MPa), L15 (59.0 ± 15.2 MPa), and L41 (35.0 ± 7.7 MPa) at 1000 °C were about 76%, 69%, and 78% of those at room temperature, respectively. A slight decline in high-temperature strength was mainly attributed to the softening of the neck between particles formed by liquid phase sintering and probably the rapidly rising temperature rate (200 °C·min^−1^) during the test procedure. 

### 3.3. Gas Permeability of Porous L-SiC Ceramics

**Figure 6:**
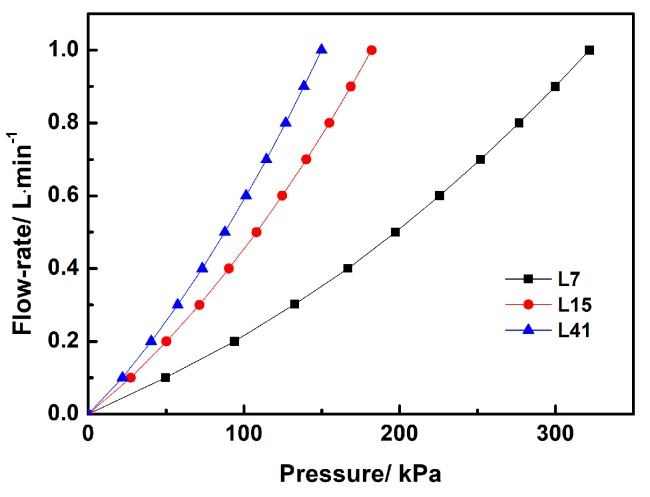
Nitrogen gas permeability of porous L-SiC ceramics with different particle sizes of initial coarse SiC powder.

Considering the above-mentioned applications of porous SiC ceramics, such as catalyst supports, gas burner media, high-temperature filters and so on, gas permeability is the key property used to measure their work efficiency. Figure 6 shows the dependence of flow-rate on nitrogen pressure and coarse particle size. The porous L-SiC ceramic with the coarse particle size of ~41 μm presented the sharpest increase in flow rate with the rising of nitrogen pressure, indicating that it had relatively high gas permeability (4.6 × 10^−14^ m^2^). The slopes of the flow-rate indicated a gradual decrease in gas permeability of the porous L-SiC ceramic with a decreased particle size of the coarse SiC. The gas permeabilities of L7 (1.4 × 10^−14^ m^2^) and L15 (2.5 × 10^−14^ m^2^) were 70% and 46% lower than that of L41. The gas permeabilities of porous L-SiC ceramics were associated with pore microstructures and their relationship can be described according to the following Carman-Kozeny’s equation:

(1) $$\mu =\frac{P_{e}D^{2}}{16f_{\mathrm{CK}}\tau^{2}}$$

where *P*_e_ is effective porosity, *D* is pore size, *f*_CK_ is Carman-Kozeny coefficient, and τ is the tortuosity of the pore. According to Equation (1), gas permeability is in direct proportion to the square of the pore size and the efficient porosity. In other words, the gas permeability was enhanced by the increased pore size and reduced by decreased efficient porosity. In this study, pore size and apparent porosity were the main influence factors on gas permeability of porous L-SiC ceramics due to their small difference in tortuosity. The result suggested that the gas permeability of porous L-SiC ceramics were in the order of μ_L41_<μ_L15_<μ_L7_, owing to the bigger numerical change ratio of the peak pore size from 3.8 μm to 1.1 μm than that of apparent porosity from 33.5% to 25.8%.

## 4. Conclusions

Porous L-SiC ceramics with excellent flexural strengths at normal and high temperature were fabricated using hot press sintering with Al_2_O_3_ and Y_2_O_3_ powder as the oxide additions. The pore microstructures and properties of porous L-SiC ceramics were tailored by altering the particle sizes of coarse SiC powders. Porous L-SiC ceramics with interconnected pores exhibited the total porosity of 25.9%–34.0%. The peak pore size of samples ranged from 1.1–3.8 μm with the increased coarse particle size. For the porous L-SiC ceramic with the coarse particle size of ~7 μm, the flexural strength was as high as 104.3 ± 7.3 MPa at normal temperature and 78.8 ± 5.1 MPa at 1000 °C. The decrease in flexural strength with the increase of coarse particle size was attributed to the bigger pore size and the concentration of stress around the contact point of the irregular pores. Moreover, the porous L-SiC ceramic with the bigger peak pore size of 3.8 μm presented outstanding gas permeability of 4.6 × 10^−14^ m^2^, even though the apparent porosity was only 25.8%. Owing to the good strength and high permeability of the as-prepared porous L-SiC ceramics, they showed commercial application potential as filters, membrane supports, and so on.

## Figures and Tables

**Table 1:** The relative densities and porosities of porous L-SiC ceramics with different particle sizes of initial coarse SiC powder.

Sample	Relative Density (%)	Total Porosity (%)	Apparent Porosity (%)	Closed Porosity (%)
L7	66.0	34.0	33.5	0.5
L15	70.1	29.9	29.8	0.1
L41	74.1	25.9	25.8	0.1
